# Apoptosis of non-parasitized red blood cells in malaria: a putative mechanism involved in the pathogenesis of anaemia

**DOI:** 10.1186/1475-2875-9-350

**Published:** 2010-12-02

**Authors:** Paulo RR Totino, Aline D Magalhães, Luciene A Silva, Dalma M Banic, Cláudio T Daniel-Ribeiro, Maria de Fátima Ferreira-da-Cruz

**Affiliations:** 1 Laboratory of Malaria Research, Instituto Oswaldo Cruz, Fiocruz, Avenida Brasil, 4365, Manguinhos, 21045-900, Rio de Janeiro, RJ, Brasil

## Abstract

**Background:**

Severe anaemia is a common complication of *Plasmodium falciparum *malaria in hyperendemic regions. Premature elimination of non-parasitized red blood cells (nRBC) has been considered as one mechanism involved in the genesis of severe malaria anaemia. It has been reported that apoptosis can occur in RBC and, consequently, this cell death process could contribute to anaemia. This study was performed to evaluate the susceptibility of nRBC to apoptosis in a malaria anaemia murine model.

**Methods:**

Balb/c mice were intraperitonially inoculated with 1 × 10^6 ^*P. yoelii *17XL parasitized RBC (pRBC) and, then, parasitaemia and anaemia were monitored. Apoptosis in both pRBC and nRBC was assessed during early and late phases of infection by flow cytometry using Syto 16 and annexin V-PE double staining and forward scatter measurement.

**Results:**

As expected, experimental infection of Balb/c mice with *Plasmodium yoelii *17XL parasites was characterized by progressive increase of parasitaemia and acute anaemia, leading to death. Flow cytometry analysis showed that a number of pRBC was in the apoptotic process. It was noteworthy that the increase of nRBC apoptosis levels occurred in the late phase of infection, when anaemia degree was notably accentuated, while no significant alteration was observed in the early phase.

**Conclusion:**

The increased levels of nRBC apoptosis herein firstly reported, in malaria infection could represent a putative mechanism worsening the severity of malarial anaemia.

## Background

Malaria remains the tropical disease of major prevalence in the world, representing great problem of public health with approximately 250 million cases and 900 thousand deaths annually [1]. From all malaria human parasites, *Plasmodium falciparum *is the most prevalent and the most frequent parasite species responsible for the severe and lethal forms of the disease. Complications associated to *P. falciparum *infection include severe anaemia, which affects mainly children and pregnant women living in malaria hyperendemic regions [2,3].

The immunological processes involved in malaria anaemia cannot be implicated as the sole cause of erythrophagocytosis during malaria [4]. It is well known that, together to mechanical rupture of parasitized red blood cells (pRBC) by the parasite and suppression of erythropoiesis, the premature phagocytosis of non-parasitized RBCs (nRBC) is also a mechanism implicated in the development of severe malaria anaemia [5,6].

Apoptosis - a physiological process of programmed cell death related to nucleated cells - can also occur in RBC as a result of intracellular influx of Ca^2+^, which leads to cell shrinkage, membrane blebbing, phosphatidylserine exposure and protease activation [7]. Although apoptosis is an essential phenomenon for cell populations regulation it was also engaged in elimination of damaged, infected and mutated cells as well as in the genesis of many disorders [8,9]. Enhanced levels of RBC apoptosis have been observed in clinical disorders in which anaemia is a common feature, such as iron and G6PD deficiency, renal insufficiency, thalassaemia, sickle-cell disease and sepsis [7] and in malaria infection apoptosis has been associated to cerebral malaria, thrombocytopenia and lymphocytopaenia [10-12]. The apoptosis of parasitized RBC does exists and it is also possible that the same phenomenon could concern normal RBC and RBC apoptosis could, therefore, contribute to the genesis of malaria anaemia. In this light, the present study was carried out to evaluate the susceptibility of nRBC to apoptosis in a murine model of malaria anaemia.

## Methods

### Experimental infection

The lethal experimental infection of Balb/c mice with *Plasmodium yoelii *17XL parasites was used as a malaria anaemia model. Where indicated, non-infected, age-matched mice were used as control. All animal experimentation was approved by the Ethics Committee on the Use of Animals of the Oswaldo Cruz Foundation (Fiocruz), Rio de Janeiro, RJ, Brazil.

For infection, female Balb/c mice aged 6-8 weeks, provided by the Centre for Laboratory Animals Breeding of the Fiocruz, were intraperitoneally inoculated with 1 × 10^6 ^*P. yoelii *17XL-pRBC in 0.2 mL phosphate buffered saline (PBS). During infection, parasitaemia and anaemia degree were routinely monitored through mice tail blood samples. Parasitaemia was determined by counting the number of pRBC in a total count of 1000 RBC in thin blood smears stained by the Romanowski's method (Panótico Rápido, Laborclin^®^, Pinhais, PR, Brazil). Anaemia was evaluated by counting the number of RBC/mm^3 ^of blood. Briefly, 2 μL of blood were suspended in 0.5 mL heparinized PBS, diluted 1:10 in the same buffer and, then, the number of RBC determined in a haemocytometer.

### Apoptosis assay

Apoptosis was identified in the early (day 4) and late (days 6-7) phases of *P. yoelii *infection through the detection of phosphatidylserine exposure (PS) at the cell surface and cell shrinkage [7]. For this propose, it was used Syto 16 and annexin V-PE double staining that identify pRBC and PS exposure, respectively. Briefly, RBC were isolated from mice tail heparinized blood by centrifugation at 350 × *g *for 10 min at room temperature, washed twice with PBS and, then, incubated at 37°C for 40 min at a density of 1 × 10^5 ^cells/400 μL in PBS containing 100 nM Syto 16 (Invitrogen). After incubation, staining buffer was discarded and the RBC were stained with 5 μL annexin V (BD Pharmingen) for 15 min at room temperature in 100 μL annexin-binding buffer (BD Pharmingen) containing 100 nM Syto 16. Finally, cells were five times diluted in annexin-binding buffer containing Syto 16 and both forward scatter and fluorescence emission were assessed using a flow cytometer (FACScalibur, Becton Dickinson)

In addition to *ex-vivo *analysis, apoptosis was also measured after incubation of RBC for 24 h at 37°C at a haematocrit of 0.5% in Ringer solution containing (in mM) 125 NaCl, 5 KCl, 1 MgSO_4_, 32 N-2-hydroxyethylpiperazine-N-2-ethanesulfonic acid (HEPES), 5 glucose, and 1 CaCl_2 _(pH 7.4).

### Statistical analysis

Statistical analyses were performed using t student test in GraphPad Prism 5.0 software (San Diego, CA, USA); a p-value < 0.05 was considered significant.

## Results and discussion

Data from experimental rodent malaria infection, widely used as malaria models, have reinforced premature destruction of nRBC as a relevant process of malaria anaemia [13,14]. Thus, to investigate the involvement of nRBC apoptosis in malaria anaemia a *P. yoelii *17XL malaria murine model was used in the present report.

**Figure 1:**
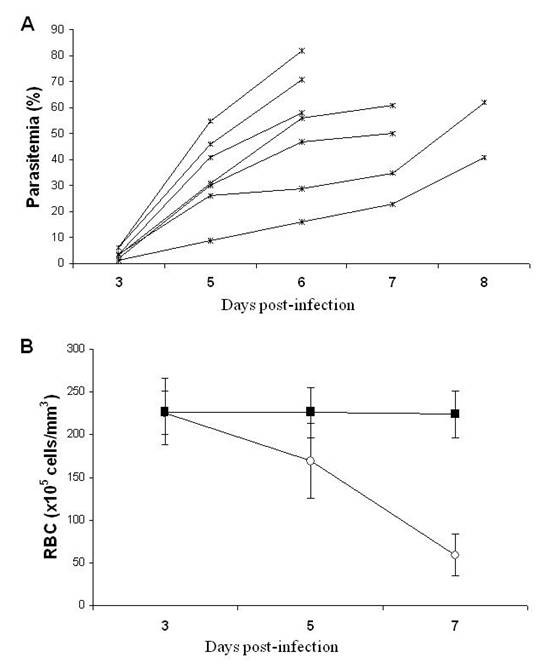
**Course of parasitaemia and progression of anaemia in *P. yoelii *17XL infected Balb/c mice**. Mice (n = 7) were inoculated with 10^6 ^*P. yoelii *17XL-pRBC and parasitaemia and anaemia degree were measured. In parallel, non-infected, age-matched mice were used as control (n = 5). (A) Individual parasitaemia in infected mice on days 3 and 5-8 post-infection. (B) Means ± SD of the number of RBC/μL of blood in infected (open circles) and non-infected (closed squares) mice on days 3, 5 and 7 post-infection. Data are representative of two separate experiments.

As expected, *P. yoelii *17XL infection was marked by an accentuated anaemia degree and it was lethal to Balb/c mice [15,16]. Infected mice started to die on day 7 post-infection with parasitaemia rates of 60,7 ± 13,3% on the day preceding the death (Figure 1A). The progression of infection was accompanied by a decline in the number of peripheral RBC, leading to acute anaemia, characterized by a drop of around 73% in the number of RBC, when compared to non-infected control mice (Figure 1B).

**Figure 2:**
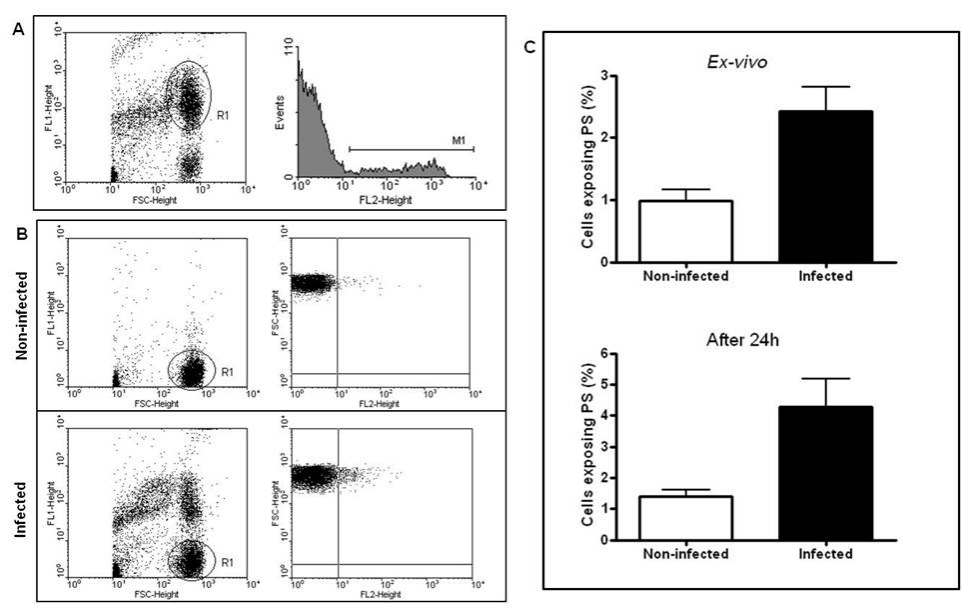
**Phosphatidylserine (PS) exposure in pRBC and nRBC during *P. yoelii *17XL infection**. Balb/c mice (n = 5) were infected with 10^6 ^*P. yoelii *17XL-pRBC and, than, PS exposure was evaluated in the late stage of infection by flow cytometry using Syto 16 (FL-1) and annexin V-PE (FL-2) dual staining. In parallel, non-infected, age-matched mice were used as control (n = 4). (A) Representative flow cytometry analysis of pRBC exposing PS in infected mice. (B) Representative flow cytometry analysis of nRBC exposing PS in infected and non-infected mice. (C) Means ± SEM of levels of nRBC exposing PS in infected and non-infected mice detected *ex-vivo *and after 24 h culture. Data are representative of three separate experiments.

**Figure 3:**
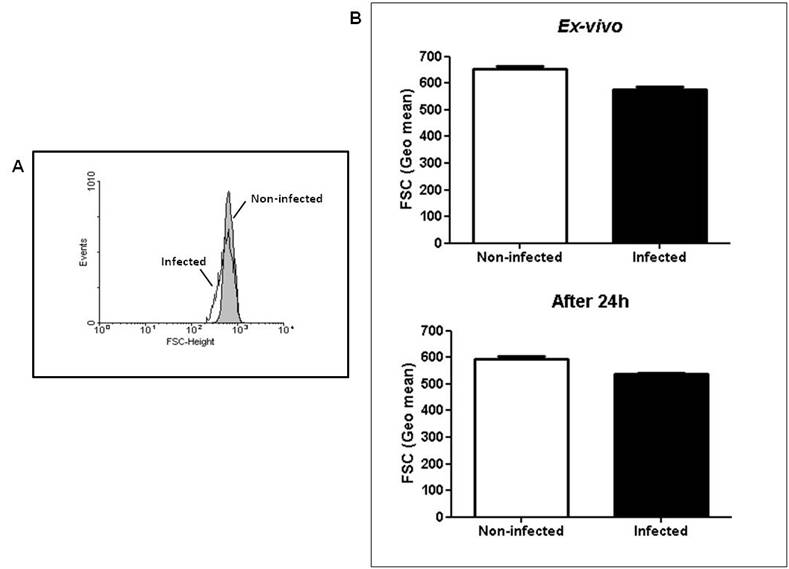
**Cell shrinkage in nRBC during *P. yoelii *17XL infection**. Balb/c mice (n = 5) were infected with 10^6 ^*P. yoelii *17XL-pRBC and, than, RBC forward scatter was determined in the late stage of infection by flow cytometry. (A) Representative flow cytometry analysis of the nRBC forward scatter in infected and non-infected mice. (B) Means ± SEM of the geometric mean of the nRBC forward scatter in infected and non-infected mice detected *ex-vivo *and after 24 h culture. Data are representative of tree separate experiments.

**Figure 4:**
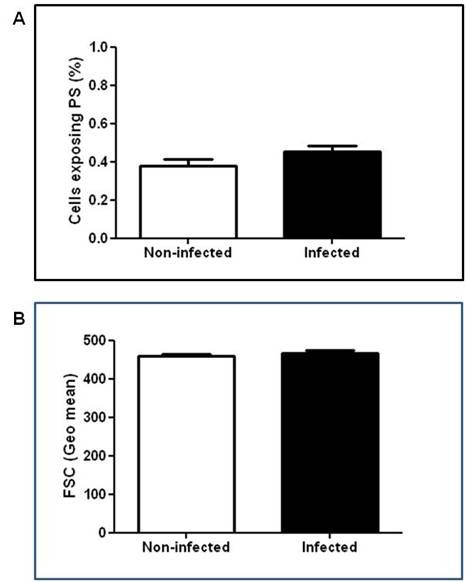
**Phosphatidylserine (PS) exposure and cell shrinkage in nRBC during early stage of *P. yoelii *17XL infection**. Balb/c mice (n = 5-7) were infected with 10^6 ^*P. yoelii *17XL-pRBC and, than, PS exposure and forward scatter was evaluated in the early stages of infection by flow cytometry. Data show means ± SEM of *ex-vivo *levels of nRBC exposing PS (A) and of the nRBC forward scatter (B) in infected and non-infected mice. Data are representative of two separate experiments.

Since pathogens have the capacity to induce apoptosis in both infected and non-infected host cells, influencing the pathogenesis of disease [8,17], it was firstly examined if the late phase of *P. yoelii *17XL infection, when anaemia degree was accentuated, was marked by RBC apoptosis. As expected, a percentage of *P. yoelii*-pRBC exposing PS and, therefore, undergoing apoptosis (Figure 2A) was observed, as reported in *P. falciparum*- and *Plasmodium berghei*-pRBC [18,19]. However, it is noteworthy that malaria infection also significantly increased the levels of nRBC exposing PS both *ex-vivo *and after 24 h *in vitro *culture, when compared with non-infected control mice (p < 0.05) (Figures 2B and 2C). Pro-apoptotic potential of malaria was further reinforced by forward scatter analysis, which showed that *P. yoelii *17XL also induced significant cell shrinkage of nRBC (p < 0.05) (Figure 3). Conversely, when both PS exposure and cell shrinkage were assessed during early phase of infection, it was not possible to identify significant alteration in the rates of apoptotic nRBC (p > 0.05) (Figure 4).

The apoptotic process in pRBC has been described as a result of intraerythrocytic plasmodia development. During schizogony, malaria parasites activate non-selective cation channel in host RBC membrane, allowing entry of Ca^2+ ^and Na^+ ^necessary for its intracellular growth [20]. Sequentially, intracellular Ca^2+ ^influx leads to activation of phospholipid scramblase and Ca^2+^-sensitive K^+ ^channels responsibles to PS exposure at outer membrane leaflet and cell shrinkage, respectively [21]. If one take in mind that apoptotic cells are quickly recognized and removed by phagocytes [22], apoptosis could act controlling parasite proliferation and even confer partial protection in sickle-cell trait carriers through accelerated clearance of ring-stage pRBC apoptosis [23].

However, if the augmented elimination of nRBC (for each pRBC, nine nRBC were removed) in both human and experimental malaria [13,24,25] could be due, at least in part, by erythrocytic apoptosis, PS exposure could also adversely promote elimination of nRBC, contributing also to malaria anaemia pathogenesis. This hypothesis could be supported by the report that deficiency of PS receptor operating in phagocytosis (i.e., CD36) can confer protection against malaria anaemia in children infected with *P. falciparum *[26].

Since nRBC apoptosis was increased during only the late phase of malaria infection, one may wonder which factors could be implicated to its induction? Notably, different types of RBC apoptosis-inducer, such as anti-RBC antibodies, oxidative stress and parasite antigens, are associated to malaria infection [7]. Indeed, anti-erythrocyte auto-antibodies and nitric oxide production are increased during plasmodia infection and they can be associated to anaemia severity [14,27,28]. Soluble factors from *P. falciparum*-pRBC are also capable of inducing apoptosis in endothelial and neuroglia cells [29] and bystander nRBC in *P. falciparum in vitro *culture undergo apoptotic process [30]. Moreover, defective production of erythropoietin - a RBC apoptosis inhibitor [31] - has been also associated with acute anaemia in malaria [32]. Thus, both host immune and parasite-derived factors, could be implicated to nRBC apoptosis during severe malaria anaemia state.

Finally, since *ex vivo *pRBC and nRBC are capable of exposuring PS and these cells were not significantly sequestrated at the present study, it is presumable that the simple presence of PS is not enough for *in vivo *endothelium adhesion, as previously suggested in *in vitro*-measured adhesiveness studies with both pRBC and nRBC [18,33,34]. Further studies on *in vivo *adherence capacity of apoptotic RBC on endothelium are necessary to clarify the role of PS, besides *P. falciparum *erythrocyte membrane protein 1 (PfEMP1), in cerebral and placental malaria pathogenesis. Intra-vital microscopy studies could help for clarifying these questions.

## Conclusion

The present report showed for the first time that malaria infection increase the levels of nRBC apoptosis, a process that could represent a putative mechanism worsening the severity of malaria anaemia. Attempts to decipher immune and parasitic factors related to RBC apoptosis during the course of malaria infection are currently underway on rodent and human malaria.

## Competing interests

The authors declare that they have no competing interests.

## Authors' contributions

PRRT participated in the design of the study, carried out the animal experimentation, flow cytometry and data analysis and drafted the manuscript. ADM and LAS helped in animal experimentation and carried out parasitaemia and anaemia measurements. DMB provided characterized parasite strains and revised the manuscript. CTDR helped in the design of the study and revised the manuscript. MFFC conceived the study, coordinated its design, and finalized the manuscript. All authors read and approved the final manuscript.
